# Next-Generation Sequencing-Based Transcriptome Analysis of *Helicoverpa armigera* Larvae Immune-Primed with *Photorhabdus luminescens* TT01

**DOI:** 10.1371/journal.pone.0080146

**Published:** 2013-11-26

**Authors:** Zengyang Zhao, Gongqing Wu, Jia Wang, Chunlin Liu, Lihong Qiu

**Affiliations:** State Key Laboratory of Biocontrol, Sun Yat-sen University, Guangzhou, China; Swedish University of Agricultural Sciences, Sweden

## Abstract

Although invertebrates are incapable of adaptive immunity, immunal reactions which are functionally similar to the adaptive immunity of vertebrates have been described in many studies of invertebrates including insects. The phenomenon was termed immune priming. In order to understand the molecular mechanism of immune priming, we employed Illumina/Solexa platform to investigate the transcriptional changes of the hemocytes and fat body of *Helicoverpa armigera* larvae immune-primed with the pathogenic bacteria *Photorhabdus luminescens* TT01. A total of 43.6 and 65.1 million clean reads with 4.4 and 6.5 gigabase sequence data were obtained from the TT01 (the immune-primed) and PBS (non-primed) cDNA libraries and assembled into 35,707 all-unigenes (non-redundant transcripts), which has a length varied from 201 to 16,947 bp and a N50 length of 1,997 bp. For 35,707 all-unigenes, 20,438 were functionally annotated and 2,494 were differentially expressed after immune priming. The differentially expressed genes (DEGs) are mainly related to immunity, detoxification, development and metabolism of the host insect. Analysis on the annotated immune related DEGs supported a hypothesis that we proposed previously: the immune priming phenomenon observed in *H. armigera* larvae was achieved by regulation of key innate immune elements. The transcriptome profiling data sets (especially the sequences of 1,022 unannotated DEGs) and the clues (such as those on immune-related signal and regulatory pathways) obtained from this study will facilitate immune-related novel gene discovery and provide valuable information for further exploring the molecular mechanism of immune priming of invertebrates. All these will increase our understanding of invertebrate immunity which may provide new approaches to control insect pests or prevent epidemic of infectious diseases in economic invertebrates in the future.

## Introduction

It is generally believed that invertebrates, including insects, lack adaptive immunity because they possess no critical cells and molecules required for adaptive immunity of vertebrates, such as T-cell, B-cell and antibodies. However, this perception changed dramatically in the last decade as phenomena similar to adaptive reactions of vertebrates have been described with many invertebrates examined. Both nonspecific [1]–[3] and pathogen-specific [4]–[7] immune responses of invertebrates were described and the phenomenon was termed immune priming [8] in order to distinguish from the adaptive immunity of vertebrates. Vaccination based mainly on the adaptive immunity has played a critical role in the prevention and control of infectious diseases of the human beings and economic vertebrates [9]. Infectious diseases has become one of the major threats to the mass rearing of invertebrates such as shrimp, crab, bees and silkworm [10]–[13], which has become booming industries in China. Understanding the molecular mechanisms of immune priming of invertebrates may provide new approaches to prevent epidemic of infectious disease in economic invertebrates mass rearing industries and resistance of insect pests against biopesticides.

The cotton bollworm *Helicoverpa armigera* is a worldwide pest that has developed strong resistance to chemical and biological pesticides [14]. This insect is a good experimental animal for the following reasons: it can be easily reared on artificial diet in the lab; the size of the larvae is suitable for processing; its biology is well understood, and the genome of the insect is currently under sequencing and will be available soon. *Photorhabdus luminescens* TT01 is the symbiotic bacteria of the entomopathogenic nematode *Heterorhabditis bacteriophora* which is highly pathogenic to many lepidoptera insects including *H. armigera* [15]. The genome sequencing of TT01 was complete in 2003 [16]. A recent study showed that the *H. armigera* larvae immune-primed with haemocoel injection of heat-killed TT01 cells resulted in a significant increase in resistance of the larvae against infection of viable TT01, and the changes on the level of protection over time after immune-priming were highly correlated to the changes in the level of major innate immune parameters, such as haemocyte density, phagocytic activity of haemocytes and antibacterial activity of cell free haemolymph (Figure S1 and S2). All these make ‘*H. armigera* + *P. luminescens*’ a good biological model for examination of the phenomena and molecular mechanism of immune priming of invertebrates.

Recently, the development of next-generation sequencing (NGS), also called high-throughput or deep sequencing technology has provided a powerful, highly reproducible and cost-efficient tool for transcriptomic research [17]. The technology has been successfully applied in many advanced research areas, including resequencing [18], microRNA expression profiling [19], DNA methylation [20], the gene expression profiles during development [21], [22] or after experimental treatments [23], gene discovery [24], [25], SSR mining [26], [27], and SNP discovery [28]–[30]. An advantage of the technology is that it could be used for gene discovery and expression profiling of organisms without reference genome by *de novo* assembly of short reads generated. Consequently, a large number of transcriptomic and genomic sequences have become available in the model or non-model organisms [31]–[37]. There are several advanced and alternative NGS platforms, such as Roche's 454 GS FLX, Illumina/Solexa Hiseq 2000 and Applied Biosystems' SOLiD [38], [39]. A full Roche/454-system run can produce about one million reads with an average read length of about 400 bp. While Solexa/Illumina or SOLiD/ABI produces nearly 20 million reads per lane, with read length ranging from 35 to100 bp, which has higher coverage and lower cost than Roche/454 [40]. The greater sequence coverage obtained contributes to facilitate the assembly of transcripts and enable rare transcripts to be identified. In absence of reference genomes, a computational *de novo* assembly approach is required, assembly efficiency and accuracy is a commonly overlooked yet critical step. The results of *de novo* assembly from short reads have been improved by using increased read length, the paired-end sequencing strategy and the development of new computational tools such as Trinity, Oases and Trans-ABySS [41]–[43]. Especially, Trinity has been developed specifically for RNA-Seq assembly using short reads and increase the applicability of Illumina sequencing and *de novo* assembly.

In this study, we employed ‘*H. armigera* + *P. luminescens* ’as a model to examine the molecular mechanism of the invertebrate immune-priming from transcriptomic views using NGS technology. Two transcriptomes of the immune-primed (TT01) and non-primed *H. armigera* larvae (PBS) were generated and compared. A comprehensive analysis of the global response to immune challenge in *H. armigera* contribute to the in-depth investigation of candidate genes involved in immune priming. The transcriptome of haemocytes and fatty body of *H. armigera* larvae obtained will be a valuable reference for future studies.

## Results

### Sequencing and *de novo* assembly of Solexa short reads

We constructed two cDNA libraries from the pooled RNA samples isolated from the hemocytes and fat body of *H. armigera* larvae immune-primed with heat-killed TT01 cells (named as TT01) and the non-primed insects (the control, named as PBS). The Illumina/Solexa NGS was used to produce 65.1 and 43.6 million clean reads from PBS and TT01 cDNA libraries, which encompassed 6.5 and 4.4 gigabases (Gb) sequencing data, respectively. All the short reads were deposited in the National Center for Biotech-nology Information (NCBI) and can be accessed in the Short Read Archive (SRA) (Accession number: SRA100105). The quality of the sequences obtained is excellent with more than 93% of reads having a Phred quality score of ≤Q20 level (error probability of 0.01). The GC contents of the PBS and TT01 libraries were 48.2% and 49.0%, respectively (Table 1).

**Table 1:** Overview of the sequencing reads.

Samples	Total Reads	Total Nucleotides (nt)	Q20 ratio (%)	N ratio (%)	GC ratio (%)
PBS	65,107,880	5,273,738,280	93.52	0.00	48.97
TT01	43,636,452	3,534,552,612	93.84	0.00	48.20

* Total Nucleotides  =  Total Reads1× Read1 size + Total Reads2× Read2 size

All clean reads from the two libraries were used for sequence assembly at the *k*-mer length of 25 using Trinity. The PBS library produced 36,365 contigs (Dataset S1) which had a length from 201 to 14,079 bp (with an average length of 990 bp and an N50 length of 1,751 bp). The TT01 library generated 31,341 contigs (Dataset S2) which ranged from 201 to 14,514 bp (with an average length of 1,025 bp and an N50 of 1,779 bp). There were no contigs shorter than 200 bp. About 34.8% (12,666) of PBS contigs and 36.4% (11,404) of TT01 contigs had a length longer than 1,000 bp.

**Figure 1:**
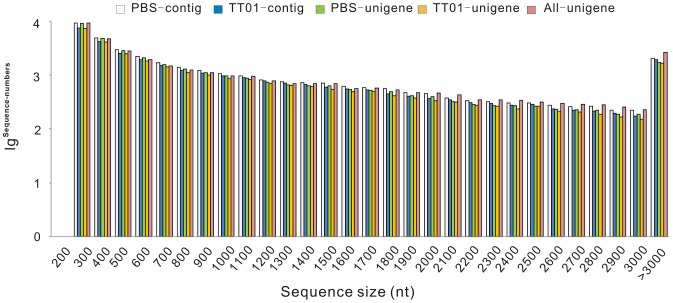
Length distribution of contig/unigene/all-unigene assembled by Trinity. X-axis represents sequence size (nt). Y-axis indicates the value of lg ^sequence-numbers^.

**Figure 2:**
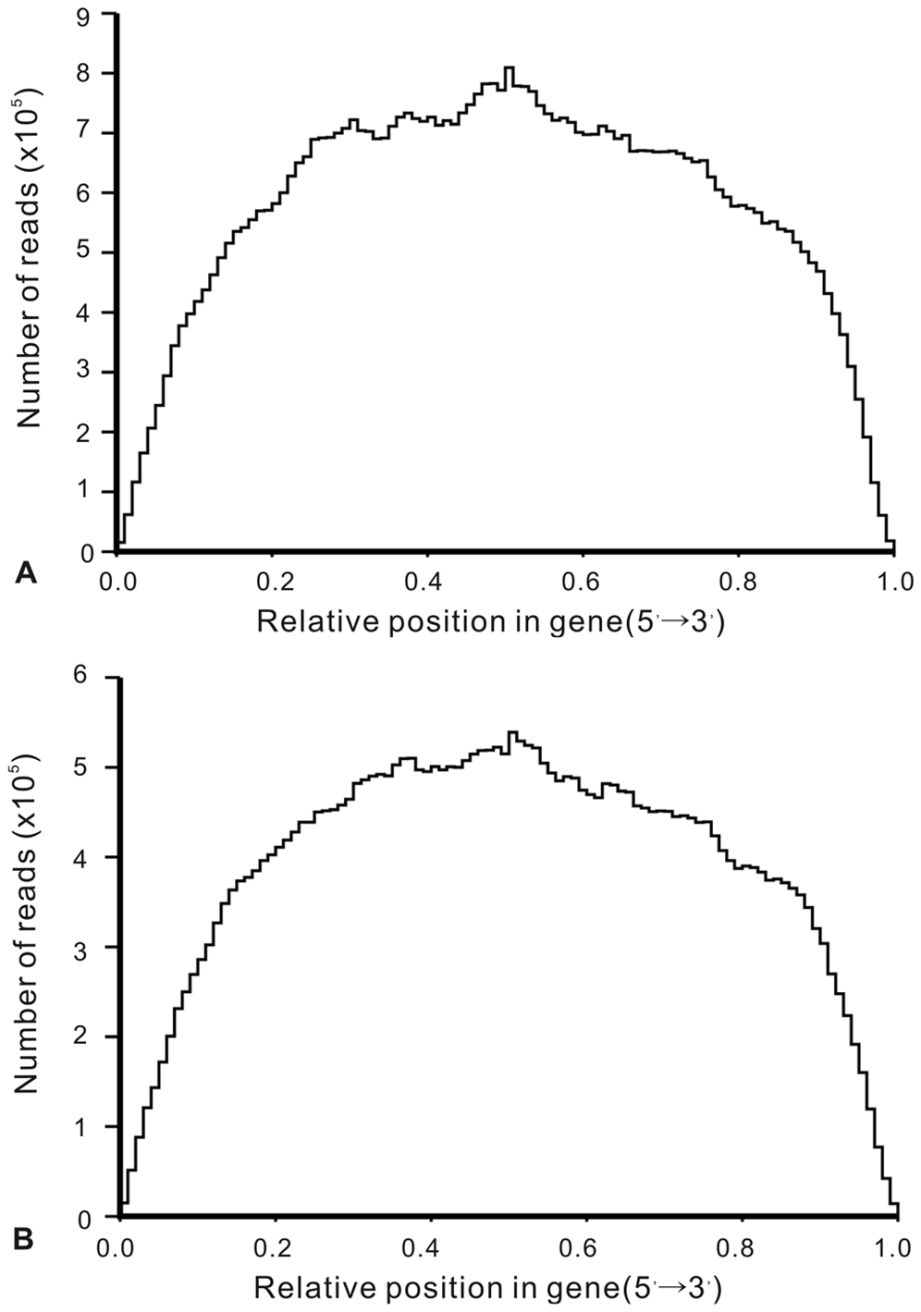
Random distribution of sequencing reads in assembled unigenes. X-axis represents relative position of sequencing reads in the assembled sequences. Orientation of assembled unigenes is from the 5′ end to the 3′end. Y-axis indicates number of reads. A: random distribution of PBS reads mapped to PBS-unigenes. B: random distribution of TT01 reads mapped to TT01-unigenes.

The scaffolds were obtained from contigs using pair-end alignment and further assembled into unigenes. As a result, 33,775 PBS unigenes (Dataset S3) were generated which had lengths varied from 201 bp to 14,079 bp (with an average of 945 bp and an N50 length of 1,678 bp). There were no unigenes shorter than 200 bp and 11,068 (32.8%) of them were longer than 1,000 bp. For TT01 library, a total of 29,485 unigenes (Dataset S4) were generated and these unigenes had an average length of 987 bp and an N50 length of 1,720 bp. There were no TT01 unigenes shorter than 200 bp either, and 10,256 (34.8%) of them were longer than 1,000 bp. TGICL [44] was used to assemble all the unigenes from both TT01 and PBS libraries to form a single set of 35,707 all-unigenes (Dataset S5) which had a length varied from 201 bp to 16,947 bp and an N50 length of 1,997 bp (Table 2). The length distribution of these sequences and the random distribution of sequencing reads in the assembled unigenes were shown in Figure 1 and 2, respectively. These all-unigenes provide a sequence basis for finding immune priming relevant genes for further studies.

**Table 2:** Summary statistics for assemblies.

Type	Sample	Total numbers	Total bases	N50 (bp)	Average length	Max length	Min length
Contig	PBS	36,365	36,011,132	1,751	990	14,079	201
	TT01	31,341	32,126,161	1,779	1,025	14,514	201
Unigene	PBS	33,775	31,918,144	1,678	945	14,079	201
	TT01	29,485	29,095,915	1,720	987	14,514	201
All-unigene	Both	35,707	38,258,496	1,997	1,071	16,947	201

### Annotation of all-unigenes

**Figure 3:**
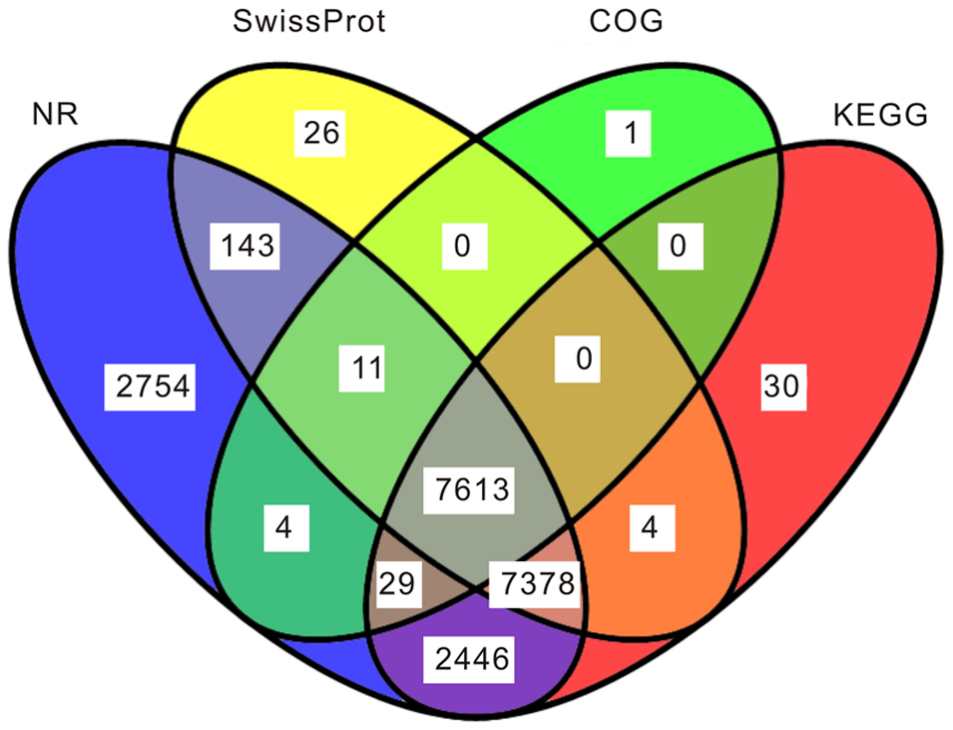
Detection of homologous genes in public databases. The numbers of annotated all-unigenes were indicated in the ellipses, respectively.

To identify the putative function of 35,707 all-unigenes (non-redundant transcripts) obtained, they were aligned using blastx to protein databases in the following priority order: NCBI non-redundant (NR), SwissProt, Kyoto Encyclopedia of Genes and Genomes (KEGG) and Cluster of Orthologous Groups (COG) with an E-value cut-off of 1e^−5^. As a result, 20,378 (57.1%), 15,175 (42.5%), 7,658 (21.4%) and 17,500 (49.0%) all-unigenes assembled by Trinity had homologous sequences in NR, SwissProt, COG and KEGG databases, respectively. Among these all-unigenes, 15,145 (42.4%) were synchronously annotated by NR and SwissProt, 17,466 (48.9%) by NR and KEGG, 14,995 (42.0%) by SwissProt and KEGG, 7,657 (21.4%) by NR and COG, 7,624 (21.4%) by SwissProt and COG, 7,642 (21.4%) by COG and KEGG, and 7,613 (21.3%) were simultaneously annotated by all four databases (Figure 3, Table S1). Also, owing to the absence of genome information of *H. armigera*, 15,269 (42.8%) all-unigenes showed no homology to known sequences deposited in these databases.

**Figure 4:**
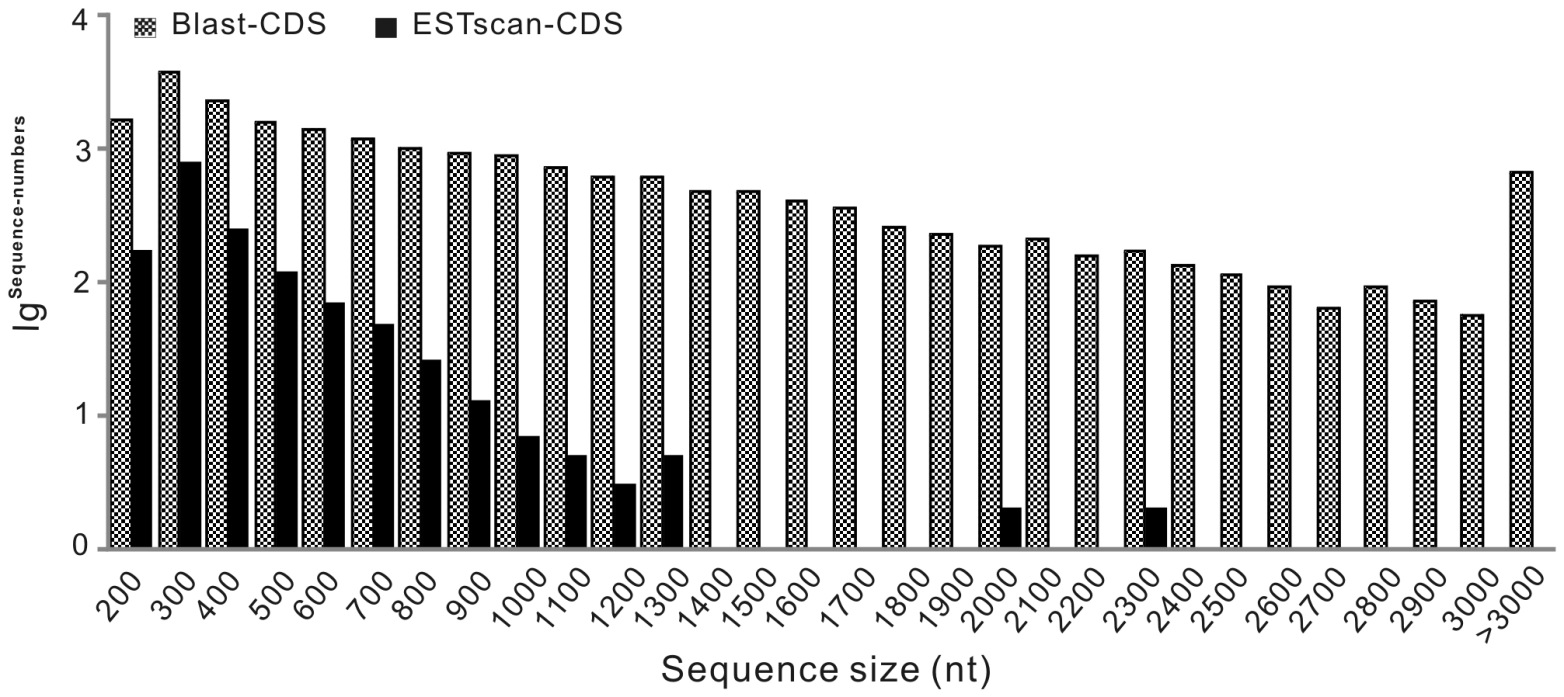
Length distribution of CDS. X-axis represents sequence size (nt). Y-axis indicates the value of lg ^sequence-numbers^. Blast-CDS represents the CDS extracted from all-unigenes using blast results information. ESTScan-CDS means the CDS predicted by ESTScan software.

**Figure 5:**
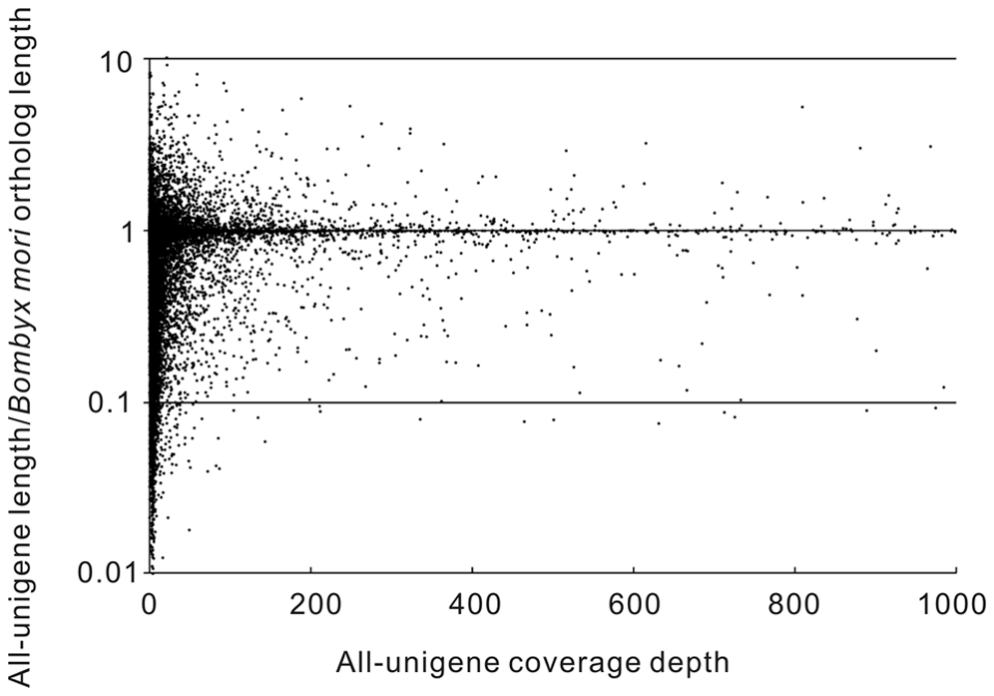
Comparison of *H. armigera* unigenes to orthologous *Bombyx mori* coding sequences. The ratio of *H. armigera* unigene length to *B. mori* ortholog length was plotted against *H. armigera* unigene coverage depth.

In addition, we extracted CDS (coding sequence) from all-unigenes using blast results information which was also used to train ESTScan, and CDS of all-unigenes have no hit in blast are predicted by ESTScan. In total, there were 20,328 blast-CDS (Dataset S6) and 1,490 CDS predicted (Figure 4, Dataset S7). Simultaneously, the extent of all-unigenes coverage provided by assembly was assessed by plotting the ratio of assembled all-unigene length to *Bombyx mori* ortholog length against coverage depth, and it was found that 31.5 % of our individual all-unigenes (11,232 out of 35,707) could map to *B. mori* (Figure 5, Table S2).

### GO classifications

**Figure 6:**
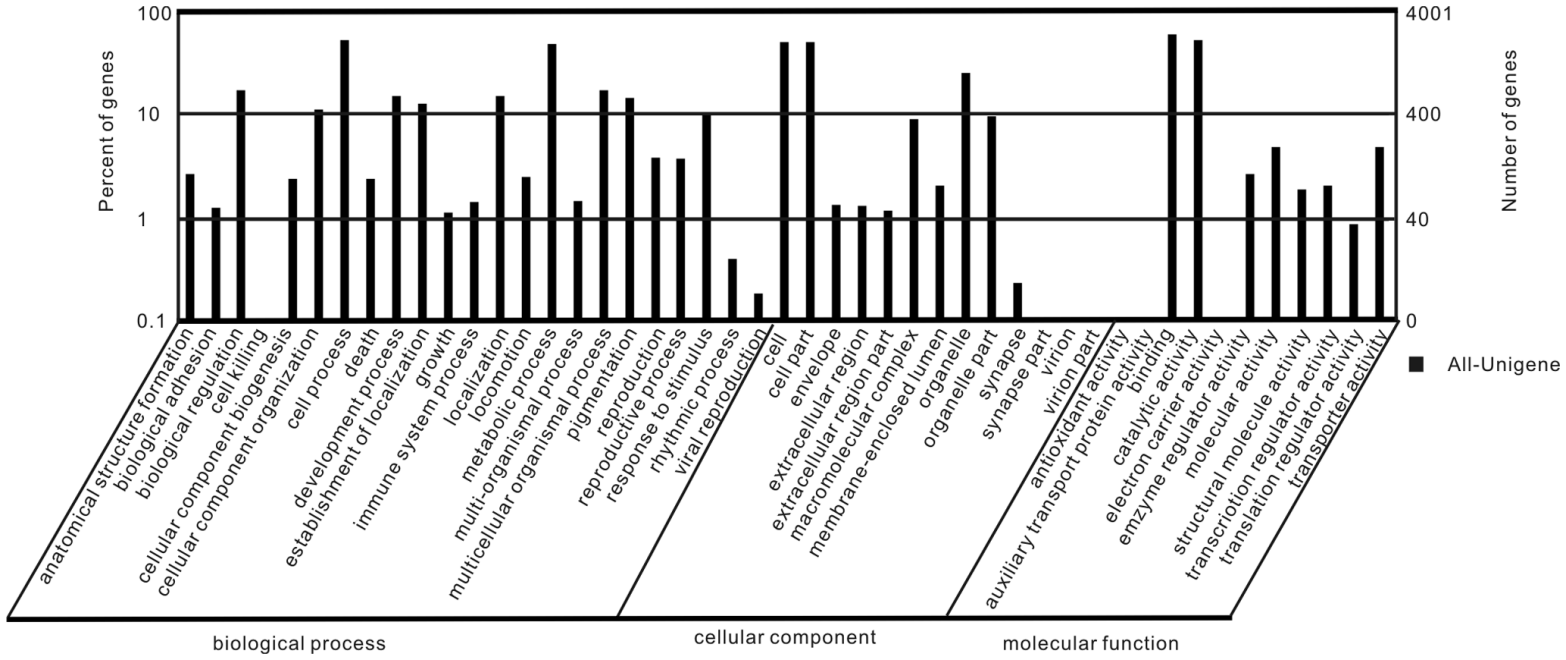
GO annotations of unigenes by Blast2GO. The x-axis indicates the subcategories, the left y-axis represents the percentage of a specific category of unigenes and the right y-axis indicates the number of unigenes.

Based on the results of the protein database annotation, a total of 4,001 all-unigenes could be assigned to 15,427 GO terms (at least one GO term using Blast2GO) and grouped into 48 functional categories (Table S3). These 4,001 all-unigenes are distributed under the three main categories of Molecular Function (3,341 all-unigenes, 5,616 GO terms), Biological Process (2,753, 6,905) and Cellular Components (1,944, 2,906), with 1,343 all-unigenes simultaneously annotated in all three categories. We observed a great number of all-unigenes from the categories of ‘Binding’ (2,242 members), ‘Cellular process’ (1,996 members), ‘Cell part’ (1,895 members) and ‘Catalytic activity’ (1,988 members), whereas fewer than 10 all-unigenes from the terms ‘Viral reproduction’, ‘Electron carrier activity’, ‘synapse part’ and ‘auxiliary transport protein activity’ (Figure 6).

### KEGG pathway mapping

KEGG pathway mapping was to identify the potential involvement of all-unigenes in biological pathways in the fatty body and hemocytes of *H. armigera*. As a result, 17,500 all-unigenes (49.0% of 35,707) matched 316,974 members involved in 267 known metabolic or signaling KEGG pathways (Table S4). The most enriched pathways included ‘RNA transport’, ‘Spliceosome’, ‘Protein processing in endoplasmic reticulum’, and ‘Ubiquitin mediated proteolysis’. These annotations provide a valuable resource for investigating specific processes, functions and pathways in the immune-priming research of *H. armigera*.

### COG classification

**Figure 7:**
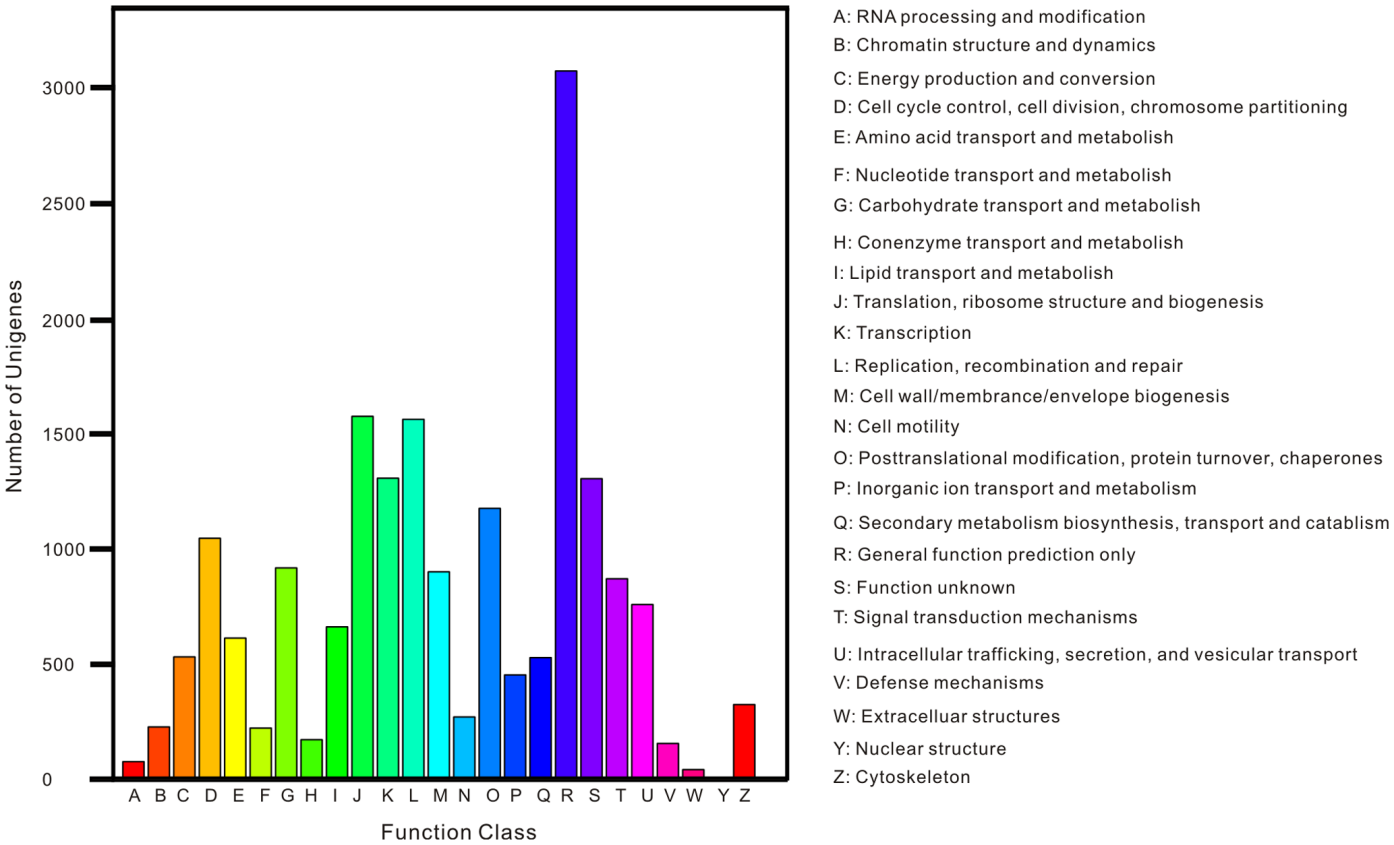
COG function classification. The capital letters in x-axis indicates the COG categories as listed on the right of the histogram and the y-axis indicates the number of unigenes.

To analyze putative protein function, all-unigenes were further annotated based on COG database [45]. A total of 7,658 all-unigenes were assigned to 83,872 COG annotations which could be grouped into 25 categories and supplied only one additional annotated all-unigene (Table S5). Among these categories, ‘General function prediction’ represents the largest group (3,080 members) followed by ‘Translation, ribosomal structure and biogenesis’ (1,579) and ‘Replication, recombination and repair’ (1,566). The category ‘Nuclear structure’ represented the smallest group (4 members). Several COG categories were probably associated with immunity, including ‘Defense mechanisms’ (157 members), ‘Intracellular trafficking, secretion, and vesicular transport’ (761 members) and ‘Cell cycle control, cell division, chromosome partitioning’ (1,049 members), etc. There were 1,308 all-unigenes belonged to ‘Function unknown’ category (Figure 7).

**Figure 8:**
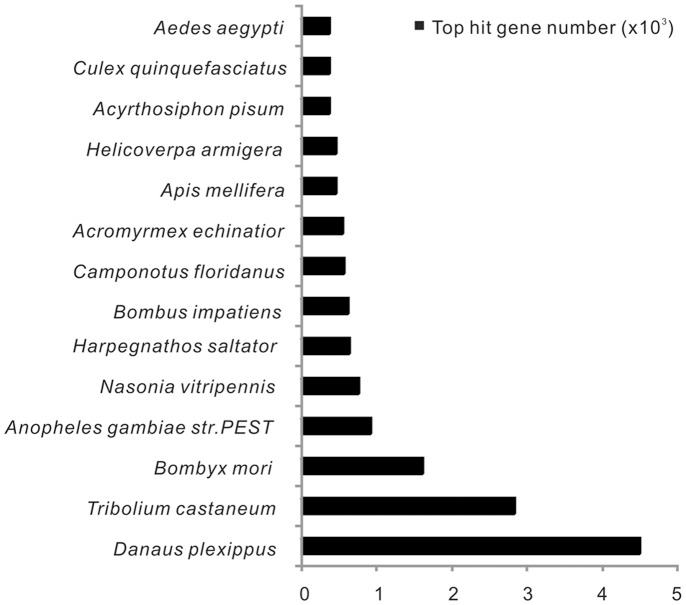
Species distribution of the annotated all-unigenes. The x-axis represents the number of gene hits and the y-axis indicates the name of insectile species in the NR database.

A total of 7,613 all-unigenes were simultaneously annotated in the four public databases examined. These annotated all-unigenes showed high homology to sequences of insect species, e.g. *Danaus plexippus* (4,509 members), *Tribolium castaneum* (2,840 members), *Bombyx mori* (1,618 members) and *Helicoverpa armigera* (466 members) according to the annotations in NR database (Figure 8, Table S6).

### Differentially expressed genes (DEGs) analysis

Analysis of the DEGs between TT01 and PBS libraries should aid our understanding of the molecular events involved in immune priming process. Gene expression levels were estimated by RPKM values. The distribution of RPKM values indicated that most of the 35,707 all-unigenes expressed at low levels. The number (%) of all-unigenes with a RPKM value <1, <10 and >100 were 18,260 (51.1%), 31,943 (89.4%) and 752 (2.1%) in PBS library; and 18,567 (52.0%), 32,012 (89.7%) and 735 (2.1%) in TT01 library, respectively.

**Figure 9:**
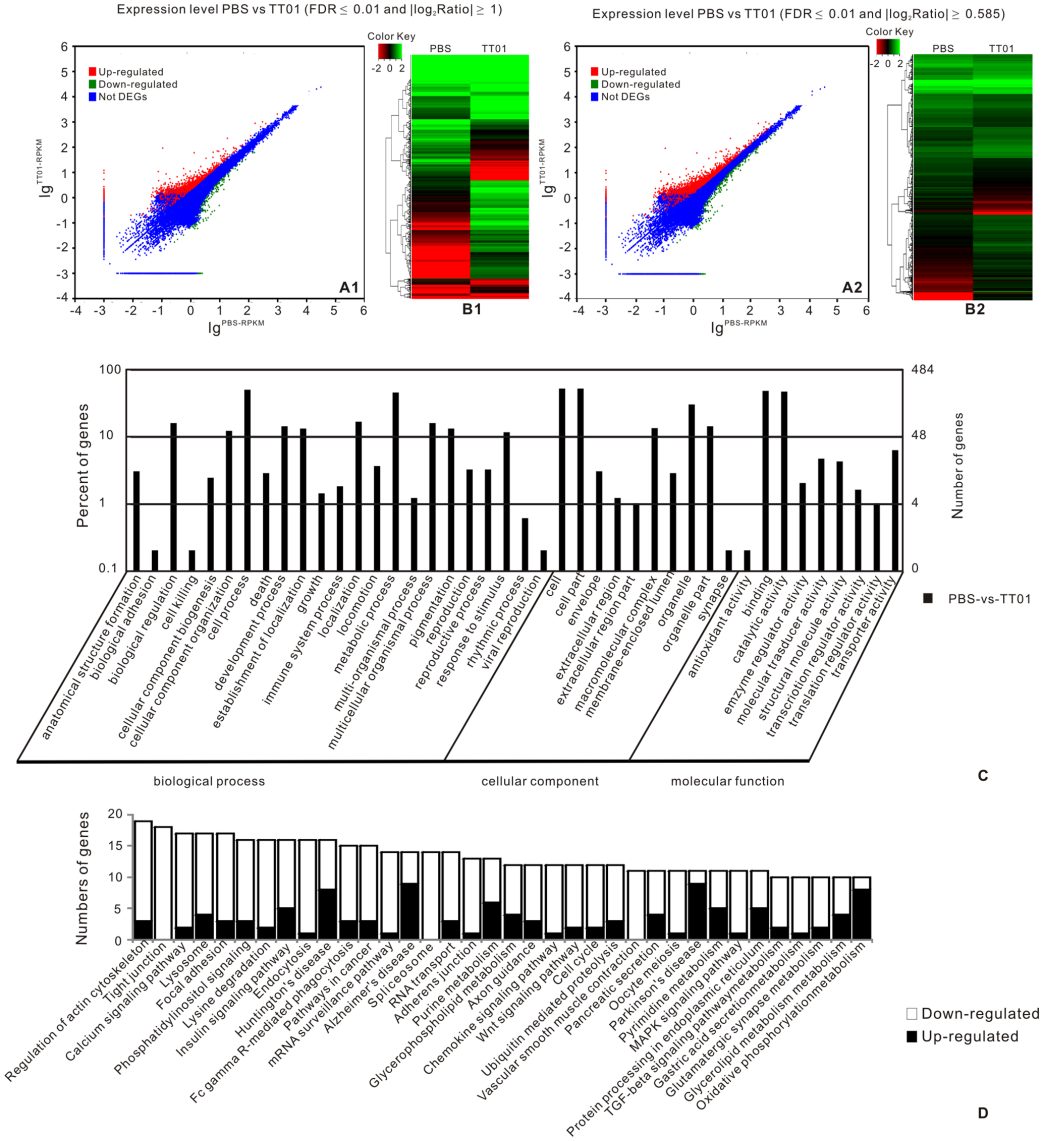
Analyses of differentially expressed genes (DEGs) between groups PBS and TT01. The graphs of A1 and A2 show DEGs (|log_2_Ratio|≥1) and DEGs (|log_2_Ratio|≥0.585). The graphs of B1 and B2 indicate the heatmap of DEGs (|log_2_Ratio|≥1) and DEGs (|log_2_Ratio|≥0.585). The graph of C shows the GO classification of DEGs (|log_2_Ratio|≥0.585) by Blast2GO. The graph of D indicates the DEGs (|log_2_Ratio|≥0.585) mapped to the KEGG pathway database.

According to the applied criteria (|log_2_Ratio|≥1, FDR≤0.001), 1,464 all-unigenes were identified as DEGs between TT01 and PBS libraries, among them 959 were up-regulated and 505 down-regulated (Figure 9 A1, Table S7). Considering that the RNA used for constructing the libraries were pooled samples collected at three time points, we set another applied criteria (|log_2_Ratio|≥0.585, FDR≤0.001) in order to reduce the dilution effect. With this criteria, 2,494 all-unigenes were identified as DEGs with 1,390 up-regulated and 1,104 down-regulated members when comparing TT01 to PBS libraries (Figure 9 A2, Table S8). Among these DEGs, we found that 1,472 (59.0%, 499 up-regulated and 973 down-regulated) members could be annotated with related databases, whereas the other 1,022 (41.0%) DEGs could not.

For every all-unigene, we used ‘gplot’ of ‘R’ to draw a heatmap applying log_A_B value (A: average RPKM of each all-unigene in different samples; B: the RPKM of the same all-unigene in one sample, Figure 9 B1 and B2) from the sequencing data. To further investigate the biological functions of DEGs, gene ontology (GO) and functional enrichment analysis were performed to map all the DEGs (|log_2_Ratio|≥0.585, FDR≤0.001) to terms in the GO and KEGG database (Table S9 and S10), and compared with the whole transcriptome background to search for genes involved in immunity, metabolic or signal transduction pathways that were significantly enriched. The result of GO enrichment analysis was presented in Figure 9 C, and the main functional groups of DEGs were related to ‘Cellular process’, ‘Binding’, ‘Catalytic activity’, ‘Cell part’ and ‘Metabolic process’, etc. The result of KEGG enrichment analysis showed that the main pathways of DEGs were significantly enriched (P-value <0.05) in ‘Regulation of actin cytoskeleton’, ‘Tight junction’, ‘Calcium signaling pathway’, ‘Cell cycle’, ‘Oxidative phosphorylation’ and ‘Phosphatidylinositol signaling system’, and also involved in ‘Lysosome’, ‘Endocytosis’, ‘ECM-receptor interaction’ and ‘Fc gamma R-mediated phagocytosis’, etc., which were correlated to immunity in spite of no significant differences in statistics (P-value >0.05) (Figure 9 D)

**Figure 10:**
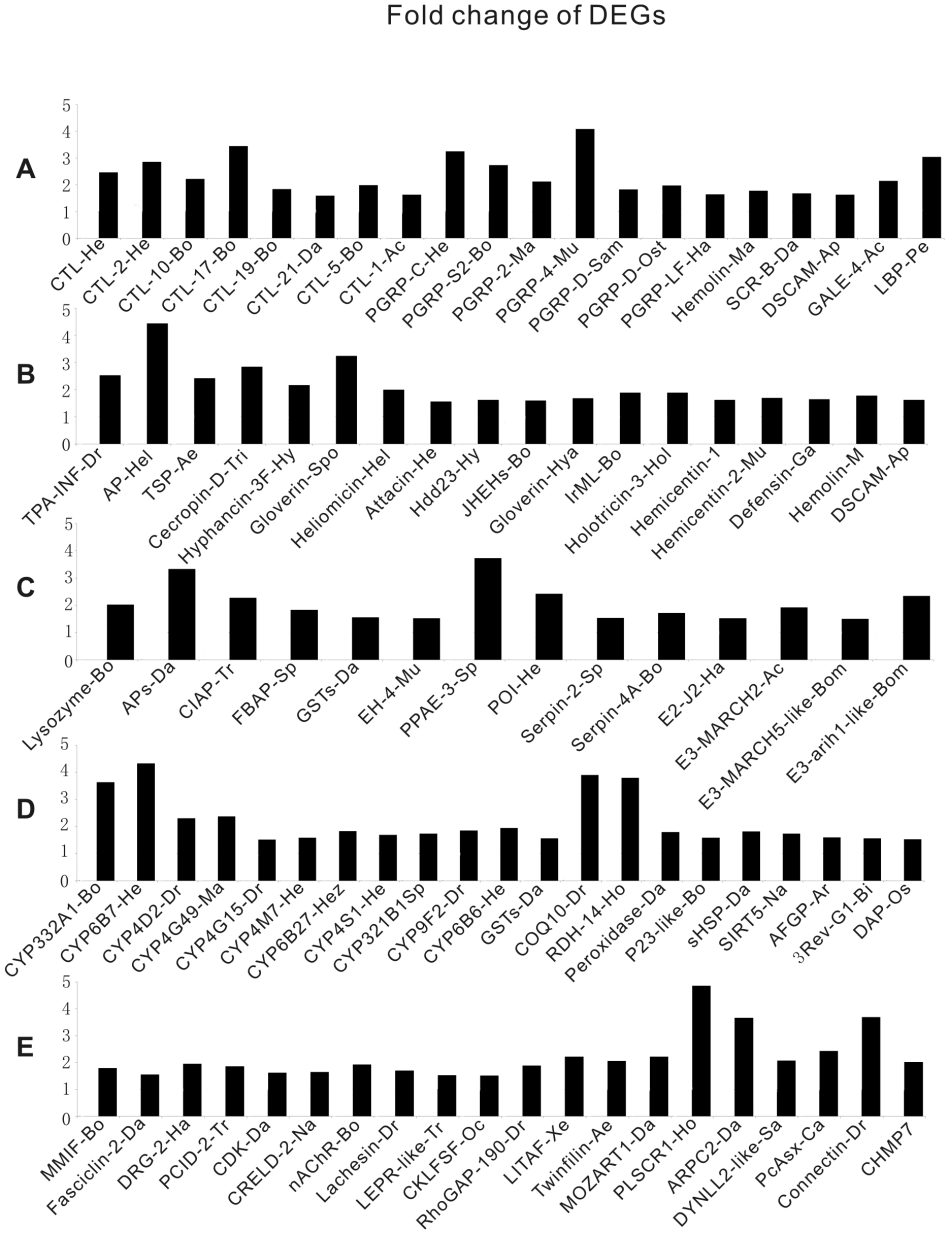
The fold change of candidate DEGs (TT01 compared to PBS). Ac: *Acromyrmex echinatior*; Ap: *Apis mellifera*; Ar: *Arctogadus glacialis*; Bi: *Biston betularia*; Bo: *Bombyx mori*; Bom: *Bombus impatiens*; Ca: *Camponotus floridanus*; Da: *Danaus plexippus*; Dr: *Drosophila melanogaster*; Ga: *Galleria mellonella*; Ha: *Harpegnathos saltator*; He: *Helicoverpa armigera*; Hel: *Heliothis virescens*; Hez: *Helicoverpa zea*; Ho: *Homo sapiens*; Hol: *Holotrichia diomphalia*; Hy: *Hyphantria cunea*; Hya: *Hyalophora cecropia*; Ma: *Manduca sexta*; Mu: *Mus musculus*; Na: *Nasonia vitripennis*; Oc: *Ochlerotatus triseriatus*; Os: *Ostrinia furnacalis*; Ost: *Ostrinia nubilalis*; Pe: *Periplaneta Americana*; Sa: *Saccoglossus kowalevskii*; Sam: *Samia cynthia ricini*; Sp: *Spodoptera litura*; Spo: *Spodoptera exigua*; Tr: *Tribolium castaneum*; Xe: *Xenopus tropicalis*; CTL: *C-type lectin*; PGRP: *Peptidoglycan-recognition protein*; SCR: *scavenger receptor*; GALE: Galectin; LBP: lipopolysaccharide-binding protein; DSCAM: Down syndrome cell adhesion molecule; AP: antibacterial protein; TSP: Thrombospondin; JHEHs: Juvenile hormone epoxide hydrolase; JHDK: juvenile hormone diol kinase; IrML: ML-domain containing secreted protein; Hdd23: immune-related Hdd23; APs: Acid phosphatase; CIAP: Alkaline phosphatase; GSTs: glutathione transferase; EH: Epoxide hydrolase; PPAE: prophenol oxidase activating enzyme; POI: phenoloxidase inhibitor protein; E3: E3 ubiquitin-protein ligase; FBAP: fat body aminopeptidase; E2: Ubiquitin-conjugating enzyme; CYP: Cytochrome P450; DAP: diapause-associated protein; AFGP: antifreeze glycoprotein; SIRT5: NAD-dependent deacetylase sirtuin-5-like; RDH: Retinol dehydrogenase; P23-like: p23-like protein; sHSP: small heat shock protein; 3Rev-G1: fatbody protein 3Rev-G1; MMIF: macrophage migration inhibitory factor; DRG: Developmentally regulated GTP binding protein; PCID: PCI domain containing protein; CDK: cyclin-dependent kinase regulatory; CRELD: cysteine-rich with EGF-like domain protein 2-like; nAChR: nicotinic acetylcholine receptor; LEPR: leptin receptor-like protein; CKLFSF: secreted protein member of chemokine-like factor super family; RhoGAP: Rho GTPase-activating protein; LITAF: Lipopolysaccharide-induced tumor necrosis factor-alpha factor homolog; MOZART1: mitotic-spindle organizing protein associated with a ring of gamma-tubulin 1; ARPC2: actin-related protein ARP2/3 complex; DYNLL2-like: dynein, light chain, LC8-type 2-like; PcAsx: Polycomb protein Asx; CHMP: charged multivesicular body protein.

Among these DEGs, we found 20 pattern recognition receptors (PRRs), 28 antimicrobial peptides (AMPs), 16 other immune proteins, 29 detoxication- and 37 cellular immunity-related proteins. The fold changes of these DEGs were shown in Figure 10 (Table S11).

### Quantitative RT-PCR (qPCR) validation of transcriptome analysis

**Figure 11:**
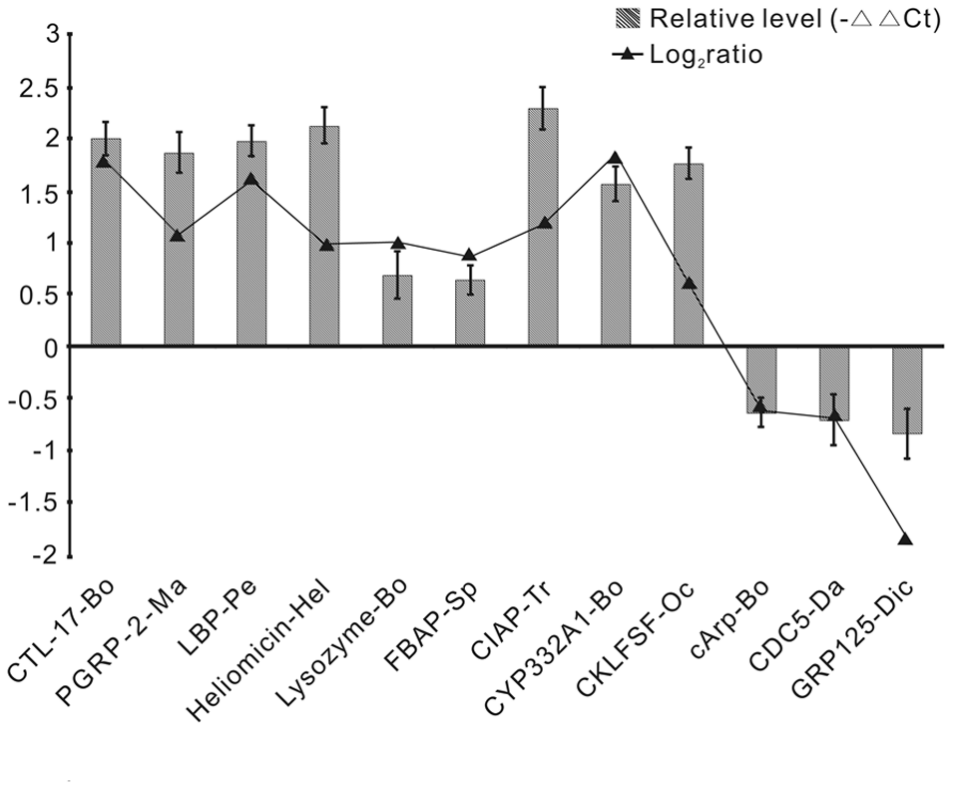
qPCR validation of the selected DEGs. The y-axis indicates the value of relative expression level (2^−△△Ct^) by qPCR and log_2_^Ratio^ of TT01/PBS by deep sequencing.

To validate our data of the deep sequencing, twelve all-unigenes were selected for quantitative RT-PCR analysis using the same RNA samples as for deep sequencing, including 9 up-regulated expression genes (CTL-17, PGRP-2, LBP, heliomicin, lysozyme, FBAP, CIAP, CYP332A1 and CKLFSF) and 3 down-regulated expression genes (cArp, CDC5 and GRP125). The qPCR results confirmed the data obtained from deep sequencing analysis and showed similar trends in up- or down-regulated unigenes (Figure 11).

## Discussion

Recently, we showed that *H. armigera* possessed a typical immune priming phenomenon which was achieved at least partly by the up-regulation of the activity and/or quantity of innate immune cells and molecules (Figure S1 and S2). The mechanism involved is not clear. Currently, there is no background genomic information available to conduct comprehensive investigations into the molecular mechanisms of this interesting phenomenon of *H. armigera*. In this study, we used the newly developed NGS technique to address this important biological question.

Two transcriptomes were generated from the major immune tissues (hemocytes and fat body) of the immune-primed (TT01) and the non-primed (PBS) *H. armigera* larvae and the DEGs were identified by comparing these two sequence data sets. We obtained 65.1 and 43.6 million high-quality clean reads from the PBS and TT01 libraries, respectively, and 35,707 all-unigenes were generated by *de novo* assembly using Trinity. For the assembled all-unigenes, 53.0% had lengths greater than 500 bp; and 48.9%, 59.7%, 44.3% and 22.3% of the annotated all-unigenes showed strong homology (E-value <1.0E^−50^) with the sequences hit in the NR, SwissProt, KEGG and COG database, respectively. Blastx analysis showed that a high proportion (57.2% of 35,707) of all-unigenes obtained had high confidence similarity to protein sequences from annotated gene catalogs of other insect species, indicating the integrity of transcript sequences assembled. The top three species with blast hits to annotated transcripts were *Danaus plexippus*, *Tribolium castaneum* and *Bombyx mori*, the insect species for which the annotations of their genomes are comprehensive and largely accepted. *H. armigera* belongs to the Noctuidae family in the Lepidopteran order and has a closer phylogenetic relationship with *B. mori* than *D. plexippus* and *T. castaneum*. The lower number of hits against the former is a result that much more *D. plexippus* and *T. castaneum* genes than those of *B. mori* are reported in the databases. All the above results suggested that the sequences of the *H. armigera* transcriptome generated in the present study were assembled and annotated correctly.

The RNA samples used in this study were a mixture of RNA isolated from two tissues collected at three time points (12, 24 and 48 hours after immune priming) at an equal ratio. Pooling is a cost-effective strategy to identify as many genes expressed in the process examined as possible [46]–[48]. However, this may lead to lost of DEGs because of dilution effect, especially those transient expressed or bidirectional regulated genes during the periods examined. In order to reduce the dilution effect of pooling strategy, we set two applied criteria for DEG identification: the frequently-applied one (|log_2_Ratio|≥1, FDR≤0.001) and a lower one (|log_2_Ratio|≥0.585, FDR≤0.001), which screened for DEGs with a change level≥2 and 1.5 folds, respectively. Consequently, 1,030 additional DEGs with 1.5–2.0 fold change were identified and among them 431 were up-regulated and 599 down-regulated. As they might not be the true DEGs, special attention was given when they were involved in the subsequent analysis.

Historically, the prevailing view in the field of invertebrate immunity was that invertebrates lack the machinery necessary for 'adaptive immunity' and rely soly on innate mechanisms which are characterised by low specificity and no memory. However, increasing empirical evidences have shaken this paradigm and showed that the immune defenses of invertebrates are more complicated and specific than previously thought [1], [49]–[52]. In a previous study, we showed that *H. armigera* larvae immune-primed with haemocoel injection of heat-killed TT01 cells resulted in a significant increase in resistance of the larvae against infection of a lethal dose of viable TT01 admitted by haemocoel injection. The immune protection level (estimated by the balance of the survival rates between the immune primed and the control groups recorded 3 days after infection with viable TT01) changed overtime post immune priming: it didn't increase significantly if infection occurred within 2 h post immunization (hpi), then rised gradually and peaked at 36 hpi; the protection levels remained high up to 72 hpi then decreased gradually. Analysis on the major innate immune parameters showed that the changes on the level of major innate parameters examined, such as hemocyte density, phagocytic activity of hemocytes, antibacterial activity of cell-free hemolymph, were highly correlated to the change in the immune protection levels of the larvae over time after immunization. Therefore, we hypothesised that the immune-priming phenomenon of *H. armigera* was achieved by the up-reguation of innate immune parameters (Figure S2). The annotated DEGs revealed in the present study supported this hypothesise:

1. It is generally believed that identification of the conserved pathogen-associated molecular patterns (PAMPs) of the pathogens by the pattern recognition receptors (PRRs) of the hosts is the first step of the innate immune-reaction process of invertebrates [53] (unlike adaptive immunity, which recognizes every antigen). Our data showed that PRRs, including hemolin, peptidoglycan recognition proteins (PGRPs), thioester-containing proteins (TEPs), lipopolysaccharide-binding protein (LBPs), c-type lectins (CTL), scavenger receptors (SCRs), galectins (GALE) [54], and down syndrome cell adhesion molecule (DSCAM) were 1.5- to 4.5-fold up-regulated in TT01 compared to PBS group. The up-regulated PRRs enabled the immune-primed insects a quicker and stronger immune responses upon a secondary infection of pathogens. The activation of the regulatory pathways triggered by the interaction between PRRs and PAMPs of the invaded pathogen, which led to the production of immune effectors of both humoral and cellular immunity.
2. In this study, we found 37 cellular immunity related DEGs (34 up-regulated and 3 down-regulated), including DSCAM, CKLFSF (chemokine-like factor super family), MMIF (macrophage migration inhibitory factor), SCR, lachesin, ubc protein, ARPC2 (actin-related protein) and PcAsx (Polycomb protein Asx), etc. These DEGs mainly mediate the phagocytosis (e.g. ubc protein), proliferation and differentiation of haemocytes (e.g. PcAsx). Consistence with this, we showed in previous studies that the hemocyte density and phagocytic activity of hemocytes of the immune-primed *H. armigera* larvae changed significantly and the pattern of change over time after immune priming was highly similar to that of survival rates (Figure S2-C, D, E). The phenomenon was reported in several other related studies. Powell *et al.* demonstrated that there was an enhancement in the phagocytic activity (expressed as number of intracellular bacteria per 100 hemocytes) of shrimp hemocytes following primary and secondary exposure to a mix of formalin-inactivated vibrios [55]. Rodrigues *et al.* suggested that an increase of granulocytes enhanced immunity of the mosquitos to bacteria, which indirectly reduced survival of *Plasmodium* parasites upon reinfection [56]. Here, we showed that transcriptional changes of the cellular immunity related DEGs were highly correlated to the enhancement of phagocytosis which contributed to a more effective antibacterial response upon reexposure to the pathogen.
3. In this study, we also detected many up-regulated DEGs related to humoral immunity, including antimicrobial peptides (such as cecropin, gloverin, attacin, defensin, hyphancin, moricin, heliomicin and hemolin) and some other immune-related proteins (such as lysozyme, lectin, acid phosphatase, alkaline phosphatase, catalase and peroxidase). This is inline with our observation that showed: 1. the cell-free hemolymph of immune-primed group had a significant increase of antimicrobial activity than that of PBS control (Figure S2-A, B); and 2. multiple differentially expressed protein bands were seen on the SDS-PAGE of cell-free hemolymph in TT01 compared to PBS group (Figure S3). All these results proved that AMPs and the immune-related proteins mentioned above were induced after immune priming, and the persistance of them in the hemolymph enhanced the protection of immune-primed larvae against the subsequent infection of the pathogen. In another word, the sequencing data provided molecular evidence of the involvement of the humoral immunity in the immune-priming at the transcriptional level.

Understanding the signal and regulatory pathways that led to the occurence of the immune priming phenomenon of the insect is the most interested issues. In the present study, several transcripts that showed similarity to those involved in the immune-related signal pathway, including the toll receptor, spaetzle, tube, ankyrin repeat domains protein (relish binding protein), COMM domain containing protein, NF-κB repressing factor and dorsal interacting protein 3 (Dip3) were differentially expressed between the TT01 and PBS libraries. Although these DEGs of critical components in the signal pathway provided valuable clues for investigating the regulatory pathways of immune priming, it is not possible to reveal the networks of these pathways based on the data obtained now because of the following reasons: 1. the primary aim of this study was to identify as many DEGs involved in immune priming as possible, therefore the whole cell of TT01 that contained many PAMPs (such as lipopolysaccharide and peptidoglycan) was injected into the insect as antigen that would trigger many signaling pathways thus increased the complexity of the study; 2. the pooling of the samples also made the work much more difficulty. Further study is needed to reveal the molecular mechanism involved in the immune priming process.

This study also detected a considerable number of up-regulated genes known to be involved in insecticide resistance or detoxification after immune priming, including cytochrome P450 (CYP), diapause-associated protein (DAP), antifreeze glycoprotein (AFGP), glutathione transferase (GSTs), small heat shock protein (sHSP), COQ10 and P23 (HSP90), etc [57]. The up-regulation of DEGs related to resistance and/or detoxification can protect the hosts from self-damage caused by the toxic intermediate metabolites produced in the process of eliminating the ‘foreign bodies’. Although immune priming is capable of enhancing the immune function of insects, it probably has an impact on the metabolism and/or development of the host due to the up-regulation of DAP and AFGP, etc. The down-regulation of many DEGs related to non-immune cellular functions detected in this study supported this view.

For 2494 DEGs identified in this study, 1,022 of them could not be matched with any existing genes. Among them, there were 176 up-regulated and 35 down-regulated DEGs with a |log_2_Ratio|≥3 and FDR≤0.001 (≥8 folds differences), indicating that these unknown genes played an important role in the immune primed host. Although the total number of up-regulated DEGs (1390) was 25.9% greater than down-regulated ones (1104), the percentage of the annotated up-regulated DEGs (499 out of 1390, 35.9%) was much less than down-regulated ones (973 out of 1104, 88.1%). For the annotated DEGs, most of the down-regulated DEGs were functionally related to general cellular process such as energy metabolism, while the up-regulated ones were immune-related, indicating that the immune related pathways of insects are poorly understood. Uncover the function of these DEGs will greatly increase our knowledge of immune priming as well as immunity of invertebrates. The sequences of these DEGs obtained set a good foundation for functional study of these genes.

## Materials and Methods

### Insects, bacteria and immunization

Insect and bacteria: The early sixth instar *H. armigera* larvae were provided by the Entomological Institute of Sun Yat-sen University. They were reared on an artificial diet at 28°C climate chambers. *P. luminescens* TT01 was preserved in liquid nitrogen. A primary form colony was inoculated into sterile LB broth and cultured at 28°C on a rotary shaker at 220 rpm until stationary stage. The bacterial cells were collected by centrifuge (10,000 g, 1.5 min) and washed three times with PBS, then another three times after the cells were killed by incubation at 100°C for 15 mins. The resulted cells were counted and diluted to 1×10^8^ cells/ml.

Immunization: The early sixth instar *H. armigera* larvae were used. For the treatment (TT01) group, 10 µl PBS solution containing 1×10^6^ heat-killed TT01 cells was injected into the haemocoel of each larva using a 50 µl micro injector. For the control (PBS) group, each larva was injected with 10 µl PBS in the same way as TT01 group. After injection, the larvae were reared individually with diet at 28°C. Twenty-four larvae from each group were taken at 12, 24, 48 hours post injection. About 30 µl of hemolymph and 200 µg of fat body was collected from each larva and preserved in Trizol in liquid nitrogen for RNA isolation.

### RNA isolation

Total RNA was extracted with Trizol (Invitrogen, USA) following the manufacturer's protocol. The concentration and quality of each RNA sample was determined using NanoDrop ND-1000 spectrophotometer (Thermo Scientific, Waltham, MA, US) and Agilent 2100 Bioanalyzer (Agilent Technologies, Santa Clara, CA, US). Equal amount of total RNA (1 µg) was taken from each sample of the same treatment group and pooled together to construct the cDNA library.

### Construction of cDNA library and illumina deep-sequencing

The samples for transcriptome analysis were prepared using Illumina's kit following the manufacturer's recommendations. Briefly, mRNA was purified from 6 µg of total RNA using oligo-dT magnetic beads. The purified mRNA was used as templates to synthesize the first-strand and the second-strand cDNA according to the protocol of Super Script Double-Stranded cDNA Synthesis kit (Invitrogen). TruSeq RNA Sample Preparation Guide was used for cutting cDNA into short fragments. After end repair and the addition of poly (A), the short fragments were ligated with sequencing adapters and enriched by PCR amplification to construct the cDNA library template. Finally, the library was loaded onto the channels of an Illumina HiSeq™ 2000 for in-depth sequencing.

### *De novo* assembly and sequence clustering

The clean reads were obtained after trimmed from raw data by removing adaptor sequences, ncRNA reads, empty reads and low quality sequences. The clean reads of PBS and TT01 libraries were assembled into unigenes using Trinity method with optimized *k*-mer length of 25, respectively. TGICL [44] was used to assemble the unigenes from TT01 and PBS samples to form a single set of all-unigenes (non-redundant transcripts). The all-unigenes assembled were aligned using blastx [58] against protein databases, with the priority order of NR (non-redundant protein database in NCBI), SwissProt, Kyoto Encyclopedia of Genes and Genomes database (KEGG) and Cluster of Orthologous Groups (COG) database (E-value≤1e^−5^). Based on the results of the protein database annotation, Blast2GO [59] was employed to obtain the functional classification of the all-unigenes based on GO terms. The WEGO [60] software was used to perform the GO functional classification for all-unigenes. ESTScan [61] software was also used to determine the directions and CDS (coding sequence) of all-unigenes that were not aligned to those in any of the databases mentioned above.

### Differentially expressed genes (DEGs) analysis

Numbers of reads per kilobase of exon region in a gene per million mapped reads were used as the value of normalized gene expression levels [17]. Differentially expressed genes (DEGs) were found out between the immune-primed (TT01) and the non-primed (PBS) libraries according to a statistical analysis of the frequency of each transcript and their corresponding P-values were performed with methods described by Audic *et al* [62]. The significance threshold of P-value in multiple tests was set by false discovery rate (FDR). We used "FDR≤0.001 and the absolute value of |log_2_Ratio|≥1" as the threshold to judge the significance of gene expression differences. Simultaneously, we set another applied criteria (|log_2_Ratio|≥0.585, FDR≤0.001) in order to reduce the dilution effect of sample pooling strategy used in this study. Gene Ontology enrichment analysis of functional significance was applied to map all DEGs to terms in the GO database, looking for significantly enriched GO terms in differentially expressed genes. For the pathway enrichment analysis, we mapped all DEGs to terms in the KEGG database and looked for significantly enriched KEGG terms. For every all-unigene, we used ‘gplot’ of ‘R’ to draw a heatmap from the sequencing data.

### Real-time quantitative RT-PCR (qRT-PCR) validation

The cDNA of 12 annotated all-unigenes was amplified by qRT-PCR to validate the repeatability and reproducibility of gene expression data obtained by RNA sequencing. Gene-specific primers were designed according to gene sequence using Primer Premier 5.0 (Table S12). The RNA samples used for the qRT-PCR assays were the same as for the RNA-Seq experiments. The first-strand cDNA was synthesized by iScript™ cDNA Synthesis Kit (Bio-Rad). The qPCR was performed using a Bio-Rad IQ5 Real-Time PCR system with SYBR-Green detection (SYBR Premix, TIANGEN) according to the manufacturer's instructions. Each reaction was run in triplicate, after which the average threshold cycle (Ct) was calculated per sample. The RPL32 gene was used to normalize expression levels, and the relative expression of genes was calculated using the 2^−△△Ct^ method.

## Supporting Information

Figure S1 **Survival rates of** ***H. armigera*** **larvae immune-primed by haemocoel injection of 10 µl PBS solution containing 1×10^6^ cells of heat-killed** ***P. luminescens*** **TT01 or** ***E. coli*** **DH5a followed by infection with a lethal dose of viable TT01 cells (200/larva) at various times post-priming.** The survival rates were scored 72 h post-infection. N: untreated control. P: PBS control (injected with 10 µl of PBS solution per larva). E: *E. coli* group (immune-primed with heat-killed *E. coli* cells). T: TT01 group (immune-primed with heat-killed TT01 cells). Values followed by different letters are significantly different (*P*≤0.05) according to ANOVA and least significant difference (LSD) test (n = 12).
(TIF)

Figure S2 **The changes on the following critical innate immune parameters of** ***H. armigera*** **larvae over time after priming with 10 µl PBS solution containing 1×10^6^ cells/larva of heat-killed** ***P. luminescens*** **TT01 or** ***E. coli*** **DH5a.** (A), Antimicrobial activity (Growth of TT01 in medium supplemented with the cell-free haemolymph; (B), Phenoloxidase activity; (C), Phagocytic rate (the number of phagocytosed haemocytes/total haemocytes ×100%); (D), Phagocytic count (the number of phagocytosed TT01 cells/total haemocytes); (E), Haemocyte density; (F), Encapsulation count. N: untreated control. P: PBS control (injected with 10 µl of PBS solution per larva). E: *E. coli* group (immune-primed with heat-killed *E. coli* cells). T: TT01 group (immune-primed with heat-killed TT01 cells). Values followed by different letters are significantly different (*P*≤0.05) according to ANOVA and least significant difference (LSD) test (n = 8).
(TIF)

Figure S3 **Protein patterns of cell-free haemolymph collected from** ***H. armigera*** **larvae at designated times after priming with 10 µl PBS solution containing 1×10^6^ cells/larva of heat-killed** ***P. luminescens*** **TT01 or** ***E. coli*** **DH5a analysed by SDS–PAGE electrophoresis and stained with Coomassie blue (n = 8).** (A), 12 to 48 h after priming; (B), 72 to 120 h after priming. P: PBS control (injected with 10 µl of PBS solution per larva). E: *E. coli* group (immune-primed with heat-killed *E. coli* cells). T: TT01 group (immune-primed with heat-killed TT01 cells). Numbers in the lower right corner of the letters represent the processing-time.
(TIF)

Table S1 **Functional annotation of all-unigenes assembled in public databases.**
(XLSX)

Table S2 **Comparison of** ***H. armigera*** **all-unigenes to orthologous** ***Bombyx mori*** **coding sequences.**
(XLSX)

Table S3 **GO classification of all-unigenes by Blast2GO.**
(XLSX)

Table S4 **The summary of all-unigenes mapping in the KEGG pathways.**
(XLSX)

Table S5 **COG functional classification of all-unigenes.**
(XLSX)

Table S6 **Species distribution of the annotated all-unigenes in NR database.**
(XLSX)

Table S7 **Summary and functional annotation of identified DEGs (|log_2_Ratio|≥1 and FDR≤0.001).**
(XLSX)

Table S8 **Summary and functional annotation of identified DEGs (|log_2_Ratio|≥0.585 and FDR≤0.001).**
(XLSX)

Table S9 **GO classification of DEGs (|log_2_Ratio|≥0.585, FDR≤0.001) by Blast2GO.**
(XLSX)

Table S10 **Summary of DEGs (|log_2_Ratio|≥0.585, FDR≤0.001) enriched in KEGG pathways.**
(XLSX)

Table S11 **Critical DEGs related to immune priming.**
(XLSX)

Table S12 **Primers for qRT-PCR validation.**
(XLSX)

Dataset S1 **Deposited PBS-contigs.**
(FA) Click here for additional data file.

Dataset S2 **Deposited TT01-contigs.**
(FA) Click here for additional data file.

Dataset S3 **Deposited PBS-unigenes.**
(FA) Click here for additional data file.

Dataset S4 **Deposited TT01-unigenes.**
(FA) Click here for additional data file.

Dataset S5 **Deposited all-unigenes.**
(FA) Click here for additional data file.

Dataset S6 **CDS of all-unigenes using blast results.**
(FA) Click here for additional data file.

Dataset S7 **CDS of all-unigenes predicted by ESTScan which have no hit in blast.**
(FA) Click here for additional data file.
