# Supplementary figures and images for: Next-Generation Sequencing-Based Transcriptome Analysis of Helicoverpa armigera Larvae Immune-Primed with Photorhabdus luminescens TT01
Source: PLoS One. 2013 Nov 26;8(11):e80146. doi: 10.1371/journal.pone.0080146 (PMC3841171; doi:10.1371/journal.pone.0080146)

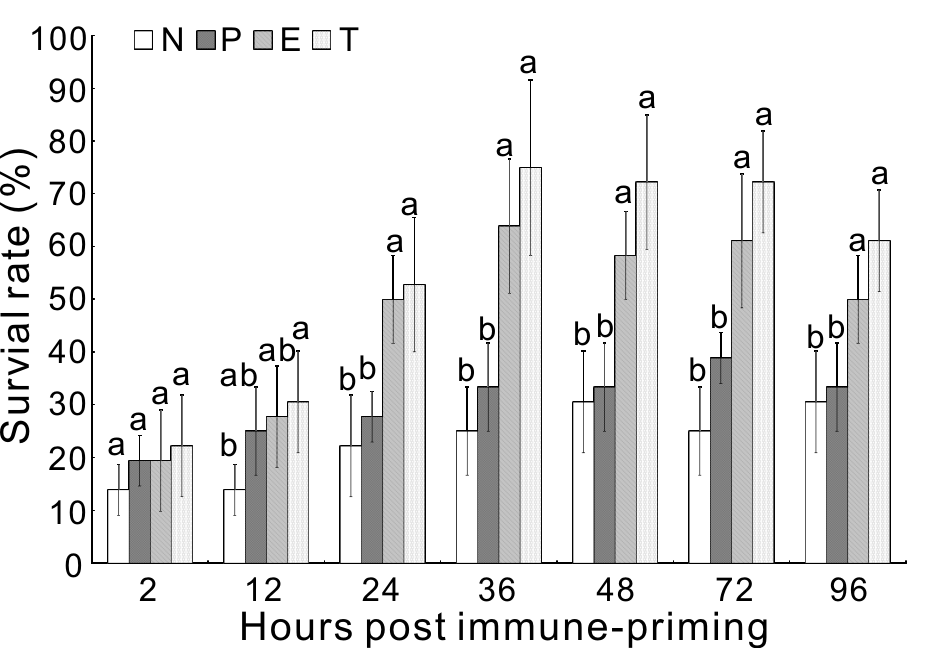

Supplement: Figure S1 — Survival rates of H. armigera larvae immune-primed by haemocoel injection of 10 µl PBS solution containing 1×10^6^ cells of heat-killed P. luminescens TT01 or E. coli DH5a followed by infection with a lethal dose of viable TT01 cells (200/larva) at various times post-priming. The survival rates were scored 72 h post-infection. N: untreated control. P: PBS control (injected with 10 µl of PBS solution per larva). E: E. coli group (immune-primed with heat-killed E. coli cells). T: TT01 group (immune-primed with heat-killed TT01 cells). Values followed by different letters are significantly different (P≤0.05) according to ANOVA and least significant difference (LSD) test (n = 12). (TIF) [file pone.0080146.s001.tif]

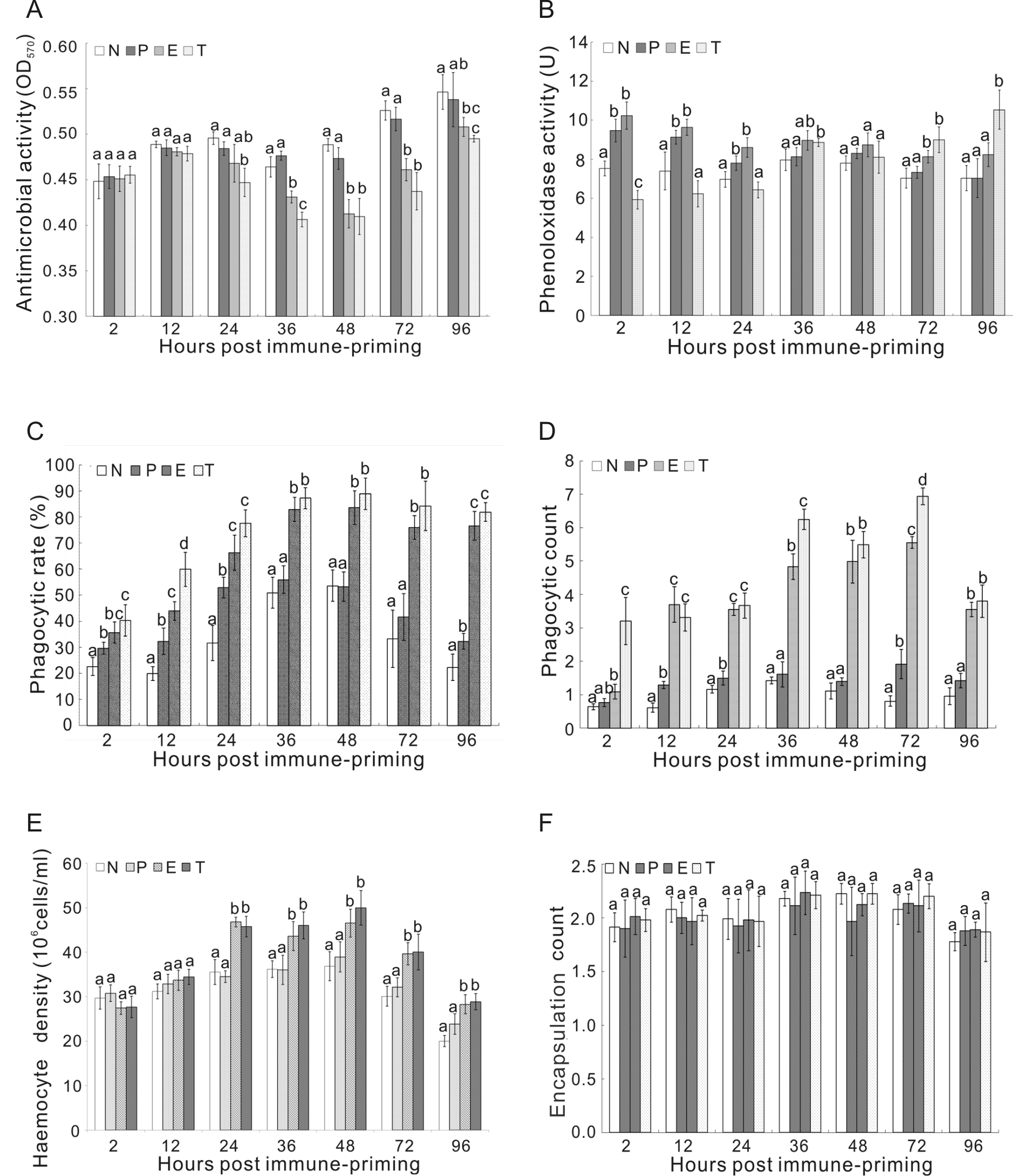

Supplement: Figure S2 — The changes on the following critical innate immune parameters of H. armigera larvae over time after priming with 10 µl PBS solution containing 1×10^6^ cells/larva of heat-killed P. luminescens TT01 or E. coli DH5a. (A), Antimicrobial activity (Growth of TT01 in medium supplemented with the cell-free haemolymph; (B), Phenoloxidase activity; (C), Phagocytic rate (the number of phagocytosed haemocytes/total haemocytes ×100%); (D), Phagocytic count (the number of phagocytosed TT01 cells/total haemocytes); (E), Haemocyte density; (F), Encapsulation count. N: untreated control. P: PBS control (injected with 10 µl of PBS solution per larva). E: E. coli group (immune-primed with heat-killed E. coli cells). T: TT01 group (immune-primed with heat-killed TT01 cells). Values followed by different letters are significantly different (P≤0.05) according to ANOVA and least significant difference (LSD) test (n = 8). (TIF) [file pone.0080146.s002.tif]

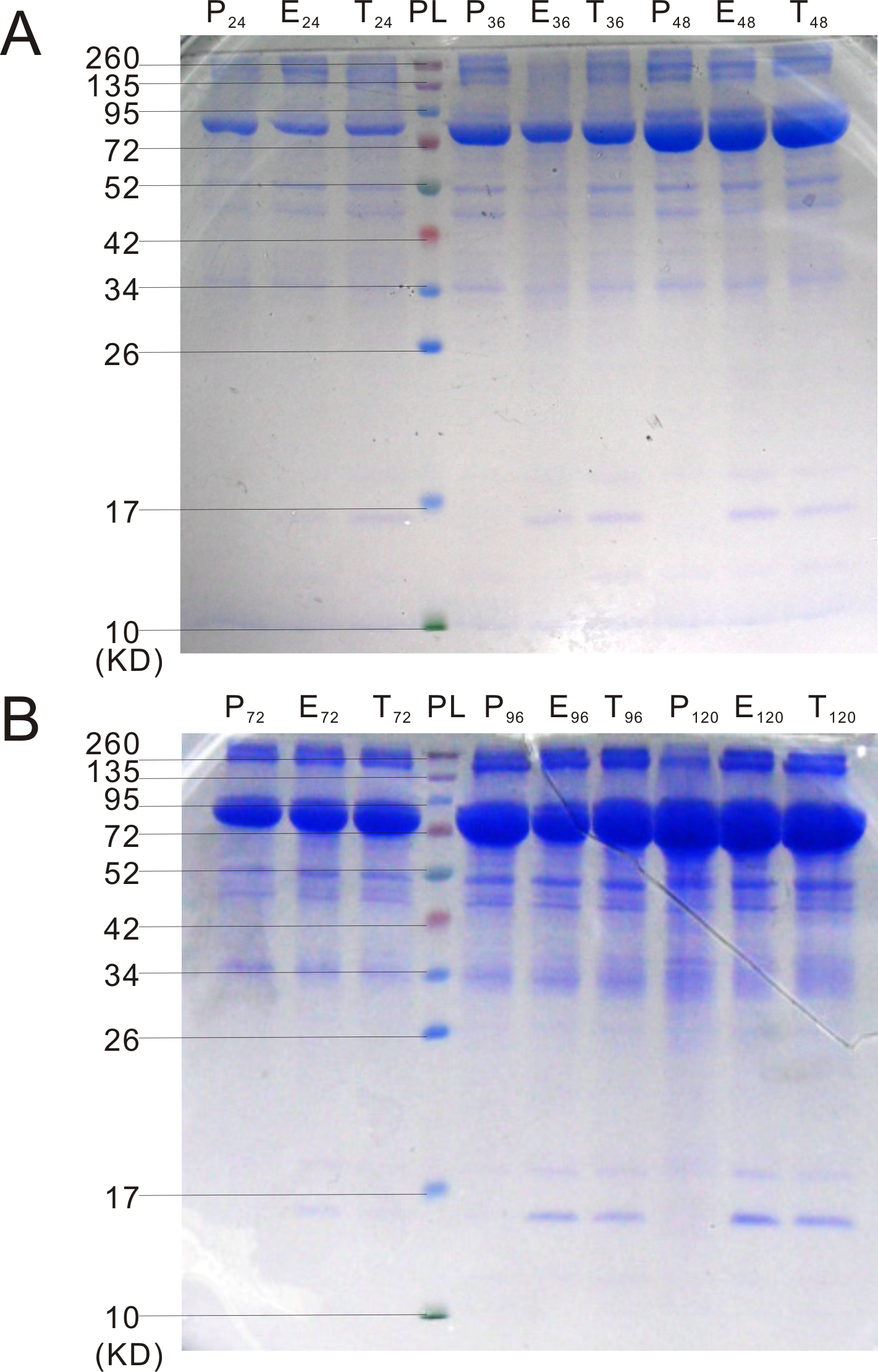

Supplement: Figure S3 — Protein patterns of cell-free haemolymph collected from H. armigera larvae at designated times after priming with 10 µl PBS solution containing 1×10^6^ cells/larva of heat-killed P. luminescens TT01 or E. coli DH5a analysed by SDS–PAGE electrophoresis and stained with Coomassie blue (n = 8). (A), 12 to 48 h after priming; (B), 72 to 120 h after priming. P: PBS control (injected with 10 µl of PBS solution per larva). E: E. coli group (immune-primed with heat-killed E. coli cells). T: TT01 group (immune-primed with heat-killed TT01 cells). Numbers in the lower right corner of the letters represent the processing-time. (TIF) [file pone.0080146.s003.tif]
